# On Pseudo Syphilis

**Published:** 1807-06

**Authors:** 


### *To the Editors of the Medical and Physical Journal.*

Gentlemen,

I am, &c.

WILLIAM NOSWORTHY.

*Crediton, Devonshire,*
*Feb.* 18, 1807.

---

### *To the Editors of the Medical and Phyſical Journal.*

Gentlemen,

HAVING for many years borne my share in the laborious profession, to the elucidation of which your valuable Journal has tended so much; my principal employment, at at present, is to learn what is going on among the rising practitioners, and particularly to attend to improvements from whatever source they may be derived. Of course, the perusal of every periodical production makes a part of my constant occupation. The first day of every month I look forward to, as the epicure does to a banquet. If the day happens to be that on which the quarterly productions arrive from the North, my festival is protracted almost to the end of the month, without reading a single article more than once over. The first of March afforded me so much amusement that I could not help expressing my gratitude to you on the occasion. Your correspondent, J. S. S. from Coventry, gives you a paper on what he calls pseudo-syphilis, a word which happily explains itself, as I believe it is not to be found in any Lexicon. In this your correspondent informs us, that after salivating his patient, finding his pseudo-syphilitic ulcer still continued, and afterwards healed without mercury, he was at a loss to account for it, till he perused Mr. Abernethy's valuable observations. Such is the sense of gratitude expressed by this writer! Now, what says another Journal, in reviewing Dr. Roberton on the power of cantharides.

If the extract there given be just, it is not easy to perceive what obligations the public owe to Mr. Abernethy. More than twenty years ago, Mr. Hunter marked several diseases which bore some resemblance to syphilis, and were exasperated by mercury, but he is much too prudent to advise us to use mercury on all these occasions. I have heard a great deal lately about these diseases resembling syphilis; and you will oblige me much, if you will allow me to request Mr. Abernethy, or your correspondent J. S. S. to inform us how we are to distinguish what is not to be confided to the powers of discrimination, "or of what consequence it is to discriminate where our practice is to be the same; and we are only to be determined by the result of experiments on our patient." It is not my wish to occupy too much of your useful pages; but I cannot help availing myself of that free discussion, which is the peculiar advantage of your Journal, to offer these doubts, institute these inquiries, and request your instruction.

I am, &c,

W. G.

*Westminster, March, 15, 1807.*

